# Supplementary material for: Asymmetric cognitive learning mechanisms underlying the persistence of intergroup bias
Source: Commun Psychol. 2024 Feb 15;2:14. doi: 10.1038/s44271-024-00061-0 (PMC11332122; doi:10.1038/s44271-024-00061-0)
Supplement: Supplementary file 1 — Supplemental Material [file 44271_2024_61_MOESM1_ESM.pdf]

Supplementary Materials

Asymmetric cognitive learning mechanisms underlying the persistence of intergroup bias

Orit Nafcha*[1,2,3], Uri Hertz*[3,4]

1. School of Psychological Sciences, University of Haifa, Haifa, Israel, 3498838
2. Translational Neuromodeling Unit (TNU), Institute for Biomedical Engineering, University of Zurich & ETH Zurich, Zürich, Switzerland, 8032
3. The Institute of Information Processing and Decision Making (IIPDM), University of Haifa, Haifa, Israel, 3498838
4. Department of Cognitive Sciences, University of Haifa, Haifa, Israel, 3498838

*Corresponding Authors:

Orit Nafcha: Department of Psychology, University of Haifa, 199 Abba Hushi, Mount Carmel, Haifa, 3498838, Israel. ornafcha@gmail.com

Uri Hertz: Department of Cognitive Sciences, University of Haifa, 199 Abba Hushi, Mount Carmel, Haifa, 3498838, Israel. uhertz@cog.haifa.ac.il

1. Supplementary Methods

Participants

N = 680, 357 Male, 316 Female, 7 Non-Binary/Prefer not to say, Age 37.2 ± 12.34

Condition	N	Age (Mean ±Std)	Male	Female	Non-Binary/Prefer not to say
All-Avoiders Group	155	40.2 ± 13.3	73	81	1
All-Avoiders Neutral	70	36.7 ± 12.7	35	34	1
All-Zappers Group	137	36.5 ± 11.8	70	66	1
All-Zappers Neutral	89	35.9 ± 12.8	54	33	1
Mixed Group	148	36.1 ± 11.2	80	66	2
Mixed Neutral	81	36.2 ± 11.4	44	36	1

Supplementary Table 1– Summary of participants' demographics by conditions

Instructions

The instructions presented to participants in the beginning of the task:

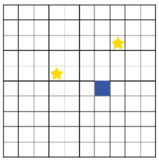

page 1:
Welcome to the Grid-World game.
In this game, you play a little square.
You live in Grid-World, looking for stars.

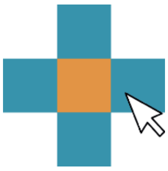

page 2:
You can use the onscreen keypad to move up, down, right or left around the grid using your mouse cursor.

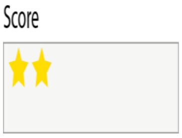

page 3:
Stars come and go – they appear from time to time, and disappear after a couple of turns if they are not collected.
The amount of stars you collected will be displayed in the 'score' window.

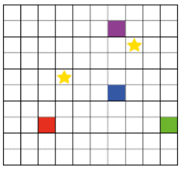

page 4:
You don't live alone in Grid-World.
The other players can also move around the grid and collect stars.

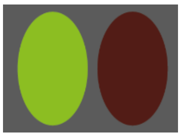

page 5:
You all take turns in moving about. The green light indicates that it is your turn to move.

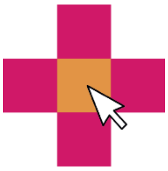

page 6:
On each turn you and the other players can choose to 'ZAP' instead of move. You 'ZAP' by pressing the orange square in the centre of the onscreen keypad, and then by choosing a direction for your ray.

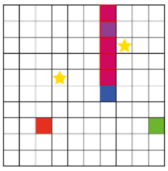

page 7:
If another player is caught in your ray, he or she will be sent to the 'Time Out Zone' for three turns.
Beware – you can also be zapped and sent to the 'Time Out Zone'.

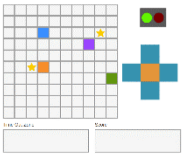

page 8:
Here is how the main game screen looks like.

page 9:
You will play the Grid World game for 100 turns with 4 other players.

page 10:
Please pick a colour for your player from the list below:

page 11:
Here is your player:
Here are the other players in the game:
Press below to start the game.

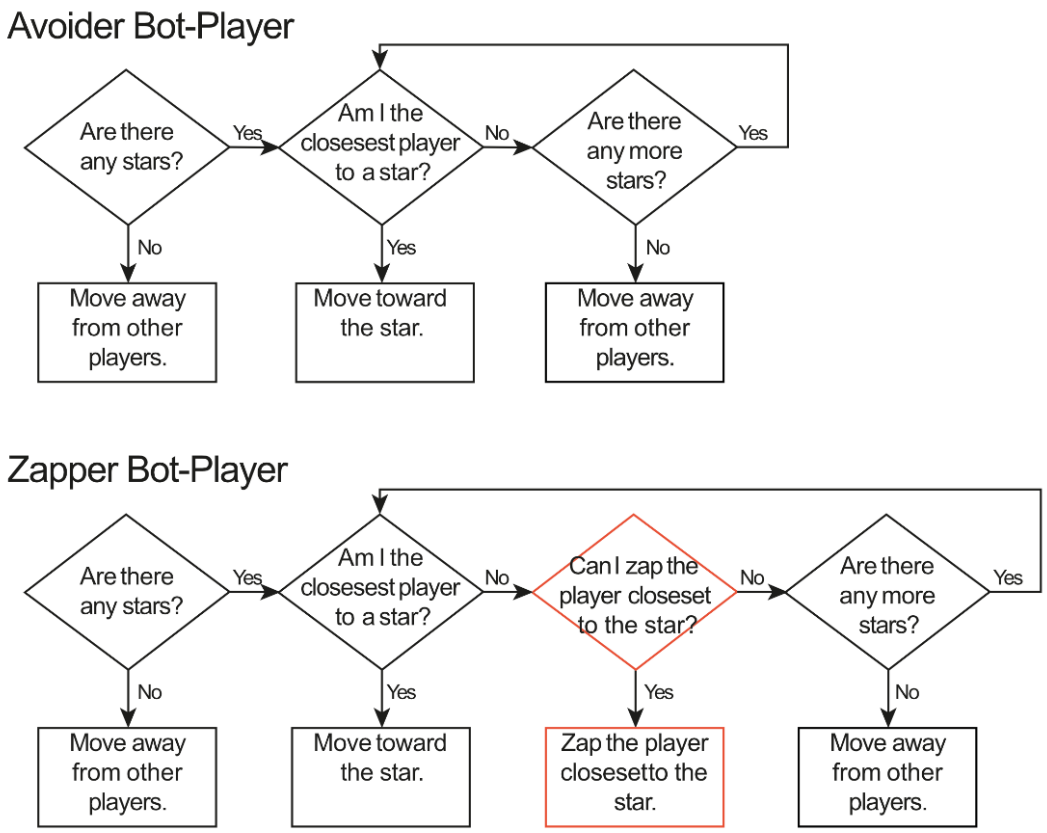

Supplementary Figure S1 – Avoider and Zapper Bot player's algorithms. Algorithms governing the zapping behavior of the zap-avoiders (top) and zappers (bottom) bot-players.

Analysis Software

All analyses were conducted with R software (R version 4.2.2). Mixed-effect linear regressions and mixed-effects logistic regressions were conducted using the packages lme4 (Bates et al. 2015) and lmerTest (Kuznetsova, Brockhoff, and Christensen 2017), Wald tests were conducted using car package (Fox and Weisberg 2019), post-hoc comparisons and estimation of marginalised means were done using emmean package (Lenth 2020), and plots were generated using ggplot2 (Wickham 2016). Power analyses were carried using R packages pwr and effectsize (Ben-Shachar, Lüdecke, and Makowski 2020). Model fitting was carried out using STAN and rstan package (Stan Development Team 2022). We also used 'rethinking' package to evaluate model fit (McElreath 2020).

Bates, Douglas, Martin Mächler, Ben Bolker, and Steve Walker. 2015. “Fitting Linear Mixed-Effects Models Using Lme4.” *Journal of Statistical Software* 67 (1). https://doi.org/10.18637/jss.v067.i01.

Ben-Shachar, Mattan, Daniel Lüdecke, and Dominique Makowski. 2020. “Effectsize: Estimation of Effect Size Indices and Standardized Parameters.” *Journal of Open Source Software* 5 (56): 2815.

Fox, John, and Sanford Weisberg. 2019. *An {R} Companion to Applied Regression*. Third. Thousand Oaks {CA}: Sage.

Kuznetsova, Alexandra, Per B. Brockhoff, and Rune H. B. Christensen. 2017. “LmerTest Package: Tests in Linear Mixed Effects Models.” *Journal of Statistical Software* 82 (13): 1–26.

Lenth, Russell. 2020. “Emmeans: Estimated Marginal Means, Aka Least-Squares Means.” https://cran.r-project.org/package=emmeans.

McElreath, Richard. 2020. *Statistical Rethinking: A Bayesian Course with Examples in R and Stan*. Chapman and Hall/CRC.

Stan Development Team. 2022. “RStan: The R Interface to Stan.” https://mc-stan.org/.

Wickham, Hadley. 2016. *Ggplot2: Elegant Graphics for Data Analysis*. Springer-Verlag New York.

2. Supplementary Analyses

The main text includes summary statistics for main analyses and does not include some of the pre-registered analyses.

Here we included full anova tables of the regressions reported in the paper, and the pre-registered analyses that were not included in the main text.

All code and data for running the analysis is included in: **https://osf.io/nx2hv/**

1. Zap Frequency Entire Block

Parameter	Sum_Squares	df	Mean_Square	F	p	Eta2_partial
BehaveHom	0.00	1	0.00	0.00	0.94	0.00
InOut	5.89	2	2.94	179.39	<0.001	0.26
Behave	1.28	1	1.28	77.84	<0.001	0.05
BehaveHom:InOut	0.03	2	0.02	1.02	0.36	0.002
BehaveHom:Behave	0.04	1	0.04	2.25	0.13	0.002
InOut:Behave	0.02	2	0.01	0.50	0.61	0.00
BehaveHom:InOut:Behave	0.07	2	0.03	2.02	0.13	0.002

Supplementary Table 2: Zap-Frequency ANOVA table

2. Zap Frequency Time-Bin

Homogeneous Conditions

Parameter	Sum_Squares	df	Mean_Square	F	p	Eta2_partial
Bin	0.56	1	0.56	9.49	0.002	0.002
InOut	2.89	1	2.89	48.58	<0.001	0.01
Behave	0.17	1	0.17	2.94	0.09	0.002
Bin:InOut	0.03	1	0.03	0.49	0.48	0.00
Bin:Behave	0.31	1	0.31	5.27	0.02	0.001
InOut:Behave	0.02	1	0.02	0.40	0.53	0.00
Bin:InOut:Behave	0.18	1	0.18	2.96	0.09	0.00

Supplementary Table 3: Zap-Frequency Time-Bin Homogeneous conditions ANOVA table

Heterogeneous Conditions

Parameter	Sum_Squares	df	Mean_Square	F	p	Eta2_partial
Bin	0.37	1	0.37	6.23	0.01	0.003
InOut	1.74	1	1.74	29.37	<0.001	0.014
Behave	0.28	1	0.28	4.66	0.03	0.002
Bin:InOut	0.02	1	0.02	0.36	0.55	0.000
Bin:Behave	0.00	1	0.00	0.07	0.80	0.000
InOut:Behave	0.05	1	0.05	0.83	0.36	0.000
Bin:InOut:Behave	0.01	1	0.01	0.19	0.66	0.000

Supplementary Table 4: Zap-Frequency Time-Bin Heterogeneous conditions ANOVA table

3. Path-Crossing

Parameter	Sum_Squares	df	Mean_Square	F	p	Eta2_partial
BehaveHom	0.03	1	0.03	4.24	0.04	0.01
InOut	0.09	2	0.05	6.65	0.001	0.01
Behave	0.03	1	0.03	4.21	0.04	0.002
BehaveHom:InOut	0.01	2	0.004	0.56	0.57	0.001
BehaveHom:Behave	0.02	1	0.02	2.31	0.13	0.001
InOut:Behave	0.01	2	0.01	0.85	0.43	0.00
BehaveHom:InOut:Behave	0.02	2	0.01	1.47	0.23	0.001

Supplementary Table 5: Path-crossing ANOVA table

4. Post-Task Star Allocation

Homogeneous Conditions

Parameter	Sum_Squares	df	Mean_Square	F	p	Eta2_partial
InOut	1547.23	2	773.62	475.91	<0.001	0.35
Behave	0.00	1	0.00	0.00	1.00	0.00
InOut:Behave	38.69	2	19.34	11.90	<0.001	0.01

Supplementary Table 6: Post-Task Star Allocation in the Homogeneous Conditions ANOVA table

Heterogenous Conditions

Parameter	Sum_Squares	df	Mean_Square	F	p	Eta2_partial
InOut	449.76	2	224.88	82.16	<0.001	0.15
Behave	120.02	1	120.02	43.85	<0.001	0.046
InOut:Behave	28.69	2	14.35	5.24	0.005	0.01

Supplementary Table 7: Post-Task Star Allocation in the Heterogenous Conditions ANOVA table

5. Post-Task Selfish Intentions Ratings

Parameter	Sum Squares	df	Mean Square	F	p	Eta2_partial
BehaveHom	3.17	1	3.17	6.84	0.01	0.01
InOut	16.53	2	8.27	17.85	<0.001	0.03
Behave	6.52	1	6.52	14.09	<0.001	0.01
BehaveHom:InOut	0.59	2	0.30	0.64	0.53	<0.001
BehaveHom:Behave	0.04	1	0.04	0.10	0.76	<0.001
InOut:Behave	4.93	2	2.47	5.33	0.005	0.01
BehaveHom:InOut:Behave	0.36	2	0.18	0.38	0.68	<0.001

Supplementary Table 8: Selfish motivation ratings ANOVA table

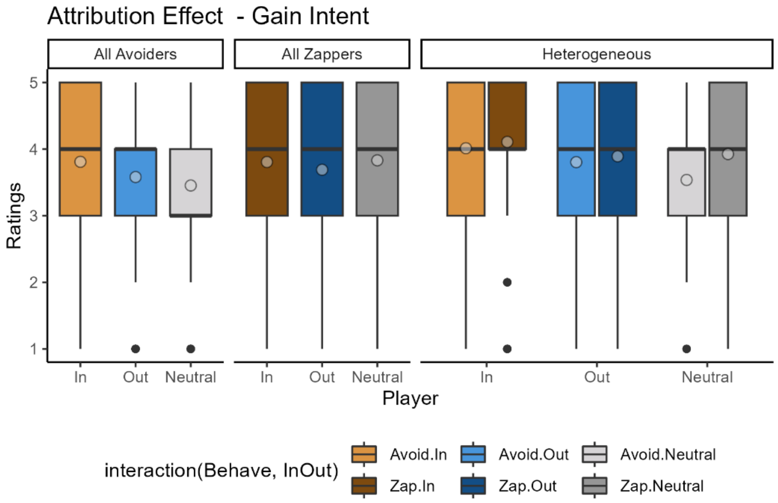

Supplementary Figure S2- Participant rated the bot-players behavior as driven by intention of gaining stars for themselves. Ratings were higher for zappers compared to avoiders, and for ingroups compared with outgroups. Boxplots include the mean by light circles, median in bold line, interquartile range is represented by the box, minimum and maximum range by the whiskers, and outliers by black dots.

6. Post-Task Harm Intentions Ratings

Parameter	Sum_Squares	df	Mean_Square	F	p	Eta2_partial
BehaveHom	35.75	1	35.75	55.12	<0.001	0.08
InOut	2.46	2	1.23	1.89	0.15	0.00
Behave	221.16	1	221.16	341.00	<0.001	0.22
BehaveHom:InOut	1.96	2	0.98	1.51	0.22	0.00
BehaveHom:Behave	59.11	1	59.11	91.14	<0.001	0.07
InOut:Behave	1.49	2	0.74	1.15	0.32	0.00
BehaveHom:InOut:Behave	5.68	2	2.84	4.38	0.01	0.01

Supplementary Table 9: Harm motivation ratings ANOVA table

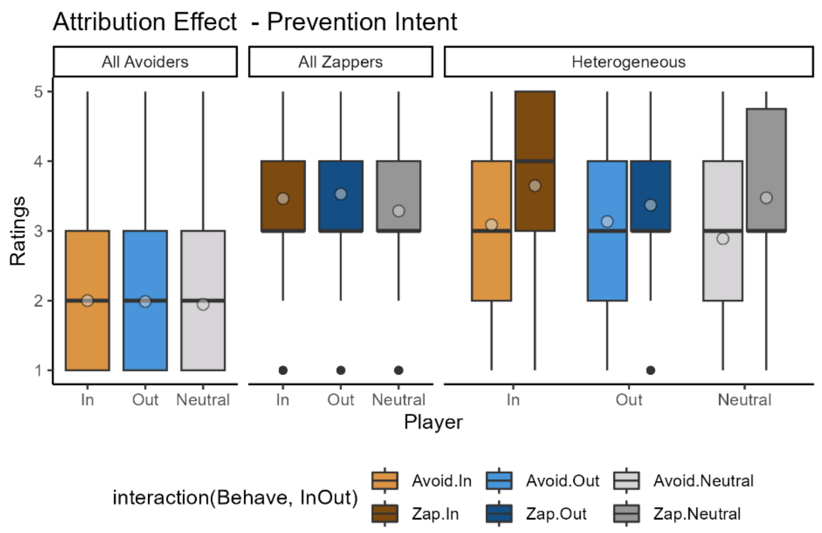

Supplementary Figure S3-Participant rated the bot-players behavior as driven by intention of preventing others from gaining stars. Ratings were higher for zappers compared to avoiders, and in the heterogeneous condition compared with the homogeneous condition. Boxplots include the mean by light circles, median in bold line, interquartile range is represented by the box, minimum and maximum range by the whiskers, and outliers by black dots.
